# Biofilm dysbiosis and caries activity: a surface or an individual issue?*

**DOI:** 10.1590/1678-7757-2023-0214

**Published:** 2023-11-13

**Authors:** Laís Daniela EV, Joice Faria POLONI, Nailê DAMÉ-TEIXEIRA, Rodrigo Alex ARTHUR, Daniela Jorge CORRALO, Sandra Liana HENZ, Thuy DO, Marisa MALTZ, Clarissa Cavalcanti Fatturi PAROLO

**Affiliations:** 1 Universidade Federal do Rio Grande do Sul Faculdade de Odontologia Departamento de Odontologia Preventiva e Social Porto Alegre RS Brasil Universidade Federal do Rio Grande do Sul, Faculdade de Odontologia, Departamento de Odontologia Preventiva e Social, Porto Alegre, RS, Brasil.; 2 Instituto Nacional de Ciência e Tecnologia – Ciências Forense Brasil Instituto Nacional de Ciência e Tecnologia – Ciências Forense, Pontifica Universidade Católica do Rio Grande do Sul, Escola de Ciências da Saúde e da Vida, Porto Alegre, Brasil.; Pontifica Universidade Católica do Rio Grande do Sul Escola de Ciências da Saúde e da Vida Porto Alegre Brasil; 3 Universidade de Brasília Faculdade de Ciências da Saúde Departamento de Odontologia Brasília DF Brasil Universidade de Brasília, Faculdade de Ciências da Saúde, Departamento de Odontologia, Brasília, DF, Brasil.; 4 Universidade de Passo Fundo Escola de Odontologia Departamento de Odontologia RS Passo Fundo Brasil Universidade de Passo Fundo, Escola de Odontologia, Departamento de Odontologia, RS, Passo Fundo, Brasil.; 5 University of Leeds School of Dentistry Division of Oral Biology Leeds UK University of Leeds, School of Dentistry, Division of Oral Biology, Leeds, UK.

**Keywords:** Dental caries, Biofilm, Microbiome, Transcriptome analysis

## Abstract

**Objective:**

This study aimed to analyze the functional profile of supragingival biofilm from sound (CAs), active (CAa), and inactive (CAi) enamel caries lesions from caries-active individuals to provide insights into the diversity of biological processes regarding biofilm dysbiosis.

**Methodology:**

A metatranscriptome analysis was performed in biofilm samples collected from five caries-active individuals. Total RNA was extracted, and the microbial cDNAs were obtained and sequenced (Illumina HiSeq3000). Trimmed data were submitted to the SqueezeMeta pipeline in the co-assembly mode for functional analysis and further differential gene expression analysis (DESeq2).

**Results:**

Bioinformatics analysis of mRNAs revealed a similar functional profile related to all analyzed conditions (CAa, CAi, and CAs). However, active and inactive surfaces share up-regulated genes (*gtsA*; *qrtT*; *tqsA*; *pimB*; EPHX1) related to virulence traits that were not overrepresented in sound surfaces. From a functional perspective, what matters most is the individual carious status rather than the surface condition. Therefore, pooling samples from various sites can be carried out using naturally developed oral biofilms but should preferably include carious surfaces.

**Conclusion:**

Metatranscriptome data from subjects with caries activity have shown that biofilms from sound, arrested, and active lesions are similar in composition and function.

## Introduction

The oral cavity is the natural habitat of a large and dynamic microbial community. Some changes in environmental conditions may result in microbial shifts related to oral diseases. Dental caries is a non-communicable disease driven by dysbiosis in supragingival biofilms due to frequent intake of fermentable carbohydrates and insufficient oral hygiene. A high-sugar diet can cause dysbiosis in the oral biofilm^1-4^ by increasing acid production from microbial metabolism and leading to tooth surface demineralization.^5^ Caries is a dynamic condition, in which lesion progression is described as caries activity. Clinically, non-cavitated lesions in progression present as whitish/yellowish opaque surfaces with loss of luster. They may have a chalky or neon-white appearance, and the surface feels rough when gently probed with a sharp instrument.^6^ Active lesions are typically located in protected sites, such as the occlusal surfaces of molars or the proximal surfaces of teeth adjacent to contact points. Arrested lesions are smooth and shiny, and the color may vary from whitish to brownish or black.^6^ It is not uncommon to observe a combination of active, arrested, and sound surfaces in a single patient. This is due to the dynamic nature of dental caries, which is a process of demineralization and remineralization that can occur over time. Individuals with active caries have a higher number of aciduric and acidogenic species within their oral microbiota.^2,7^ It is known that microbial composition and gene profile of dental biofilm change according to lesion severity.^8^ However, it is unclear whether microbial dysbiosis occurs in biofilms within the whole mouth or is localized and specific to biofilms present at an active demineralized surface. Some studies assessed dental biofilm’s microbial and functional profile by pooling samples from different oral surfaces and considering them as part of the whole mouth microbiome. Dysbiosis is likely to occur similarly in all hard tooth surfaces, so it would be reasonable to pool biofilm samples from similar tooth surfaces. However, if the biofilms from the surfaces of active carious lesions differ from the sound ones within an individual, then the biofilms should be assessed specifically at the tooth site. These questions can be addressed by characterizing the composition and functional profile of the supragingival biofilm according to well-defined clinical status at the tooth surface level.^9^ In this way, metatranscriptome analysis can provide valuable knowledge about gene content, functional profile, and genetic variability on complex microbial samples, such as those found on tooth surfaces.

Our previous study^10^ described the operational taxonomic units related to active, inactive, and caries-free individuals. This taxonomic analysis at the genus level revealed that the supragingival bacterial communities presented intra-individual similarities. However, inter-individual diversity and differences in bacterial composition showed that the subject’s caries activity status matters more than the clinical condition at the tooth site level.^10^ This study aimed to understand the differential gene expression of the functionally active microbiome of supragingival biofilm collected from active non cavitated lesions (CAa), from inactive non-cavitated lesions (CAi), and sound (CAs) surfaces of caries-active individuals to provide insights on the diversity of biological processes during caries progression. Longitudinal clinical studies by our research group have already demonstrated that the patient’s caries activity status determines the risk of lesion development, regardless of the surface state (sound, inactive, or active).^11^ Therefore, we hypothesized that the biofilm from different carious conditions in the same patients presents a similar functional profile.

## Methodology

### Study design

The data used in this work is a subset sampling from a previous study,^10^ carried out at the Biochemistry and Microbiology Oral Laboratory (LABIM/UFRGS) and at the Division of Oral Biology Laboratory, University of Leeds, United Kingdom, approved by the UFRGS Ethics Committee (CAAE 56583316.8.0000.5347). This study included only samples from caries-active volunteers (CA, n=05). The oral health status of each individual was evaluated at baseline by a dentist following the Nyvad’s criteria and using the visual-tactile method. The diagnostic exam was performed using dental equipment, artificial light, dental isolation (cotton rolls), and teeth air-drying. The lesion criteria were: (1) Active Non-Cavitated Caries Lesions (ANCL): whitish/yellowish opaque surface with loss of luster, exhibiting a chalky or neon-white appearance; the surface felt rough when the tip of a sharp probe was moved gently across it;^2^ Inactive Non-Cavitated Caries Lesions (INCL): shiny and felt smooth surface on gentle probing, and color varying from whitish to brownish or black.^6^ The inclusion criteria were not using antimicrobial agents (intravenous, muscle, or oral route) for at least two months before sample collections and not being under orthodontics treatment. All subjects provided informed consent or assent for participation (Resolution 466/2012, National Commission of Ethics in Research, National Council of Health, Brazil). All patients received dental treatment at UFRGS.

### Samples collection and storage

After baseline examination, subjects were recommended to avoid using topical antimicrobial agents and to return one week later to collect dental biofilm. The volunteers were asked to refrain from brushing their teeth for at least 12 hours and eating for at least two hours before sampling.

Supragingival biofilm (SB) samples were collected with sterilized Gracey curettes, one for each collection surface, described in Table 1. Samples from three different surfaces were collected of each patient: 1) CAa samples: all ANCL sites; 2) CAi: all INCL sites; 3) CAs: at least four sites of sound dental surfaces.

**Table 1:** Sociodemographic description of the samples

	P1	P2	P3	P4	P5	Mean (Std. deviation)	Median (percentile 25; 75%)
Sex	Male	Female	Female	Female	Female		
Age	32	12	60	14	31	29.8 (19.27)	31 (14; 32)
DMF-T (Decayed, Missing, and Filled Teeth)	19	20	24	12	16	18.20 (4.50)	19 (16; 20)
DMF-S (Decayed, Missing, and Filled Surfaces)	21	31	83	39	46	44.00 (23.70)	39 (31; 46)
PI (Plaque Index)	25%	27%	5.4%	26,10%	34.5%	23.60 (10.84)	
GBI (Gingival Bleeding index)	1%	9.6%	3.6%	3%	18%	7.04 (6.92)	
Sucrose consumption between meals	1	1.5	1	1.5		1.25 (0.29)	1 (1; 1)
Self-reported Frequency of Oral Hygiene per day	2	2	3	4	6	3.4 (1.67)	3 (2; 4)
Professional Fluoride	Yes	No	No	Yes	Yes		
Cavitated lesions	No	No	No	Yes	No		
Surface of collection							
Active lesions	Buccal (18, 17, 27, 38, 46, 48)	Occlusal (17, 27, 37)	Buccal (23)	Occlusal (14, 15, 35, 34)	Occlusal (28, 38, 35, 48)		
Inactive lesions	Buccal (13, 14, 16)	Buccal, Approximal (15,14,12,11, 21, 22, 24, 25)	Distal (45)	Occlusal (24, 25, 26)	Occlusal (17,15,25,37, 36,47)		
Sound surfaces	Buccal (21,11,33,32, 31,41,42,43)	Approximal, Lingual (36, 46, 47)	Buccal (13)	Approximal (32, 31, 41, 42)	Approximal (33, 32, 31, 41, 42, 43)		

The samples were immediately stored in 1 mL RNA stabilization solution (RNAlater™ Solution, Invitrogen, Thermo Fisher Scientific Baltics UAB, Vilnius, Lithuania) at room temperature for overnight RNA stabilization. After that, all samples were centrifuged (10,000 rotations per minute for 5 minutes) for sedimentation of all biofilms and removal of the RNAlater™ solution, and stored in a −80ºC freezer (Thermo Scientific™ Forma™ 88000 Series −86°C Upright Ultra-Low Temperature Freezers, Thermo Fisher, Leicestershire, UK) for RNA preservation.

### RNA Extraction and Quantification

UltraClean^®^ Microbial RNA Isolation kit (Qiagen) was used for total RNA extraction, according to the supplier protocol, with prior treatment steps with Lysozyme (for 10 minutes; 37ºC) and DNase I (on-column protocol). RNA quantification was performed using the Quant-iT™ RiboGreen^®^ RNA Reagent and Kit (Invitrogen, Ltd.), and samples with more than 30 ng of RNA were used for the following steps.

### Library preparation and RNA-sequencing

Genomic library preparation was performed with True Seq^®^ Sample Preparation Guide, Low Sample (LS) Protocol Illumina (Illumina, Inc., San Diego, CA). The library preparations follow the steps: RNA fragmentation; cDNA repair; adenylation of 3’-end; adaptor ligation; fragment purification; DNA enrichment. The quality of the libraries was checked with Agilent Technologies 2200 Tapestation and classified as good quality libraries for sequencing with at least 269 base pairs (bp). The dsDNA (double-strand DNA) was quantified with Quant-iT™ PicoGreen^®^ dsDNA Kit (Turner BioSystems, Inc., CA) and normalized before sequencing. Sequencing was performed in Illumina HiSeq 3000 (Illumina Inc.) for paired-end sequences of 150 base pairs (2x150 bp).

### Data treatment

The quality of the raw data was checked by the FastQC quality control tool (Babraham Bioinformatics Institute). The first evaluation of raw reads exhibited a good average quality, indicating only the presence of adapters and few sequences with short length. To improve the quality of the reads, *Trimmomatic* software^12^ was used with three parameters: ILLUMINACLIP (remove overrepresented sequences and adapters); SLIDINGWINDOW 4:15; MINLEN75. After trimming, 91.98% of the raw data remained.

The trimmed data were submitted to *SortMeRNA* program to remove the rRNA sequences (parameters: fastx, num_aligments1, paired_out, log, SILVA database).^13^ After sRNA depletion, the remaining reads were aligned to the human genome (GRCh38) to remove host sequences using the Hisat2 program (Hisat2_2.2.1), and non-aligned reads were assumed to be microbial.^14^ The resulting data were submitted to the SqueezeMeta (SQM) pipeline for metatranscriptome analysis.^15^ The samples were submitted together in co-assembly mode, generating three SQM objects: CAa, CAi, CAs. SQM pipeline includes: contigs assembly with *Megahit* software;^16^ gene prediction with *Prodigal* software;^17^ taxonomic assignment with *Diamond*;^18^ “binning” assignment with *MaxBin* software;^19^ quality of bins was checked with *CheckM*.^20^

### Differential Gene Expression Analysis

To estimate differential gene expression, the quantified mapped reads resulting from the SqueezeMeta pipeline were used as input in DESeq2 R packages.^21^ Differentially expressed genes were selected by considering FDR<0.05 and |log2FC|≥1.

Principal Components Analysis (PCA) was constructed with Factoextra R package and the heatmap plot was generated using pheatmap R package. The correlation coefficients were performed with Hmisc R package*.* Venn Diagrams were constructed online.

## Results

The clinical status of the individuals enrolled in this study is shown in Table 1, which describes that the patients were mainly female, with ages ranging from 12 to 60 years. The mean DMF-S (considering the “D” component surfaces with non-cavitated and cavitated lesions) was 44.0. The plaque and gingival bleeding indexes suggest that, although patients had active-caries lesions, their oral hygiene was acceptable. Except for two patients, all had undergone professional fluoride treatments before the biofilm collection. A single patient also presented cavitated lesions.

**Figure 1:**
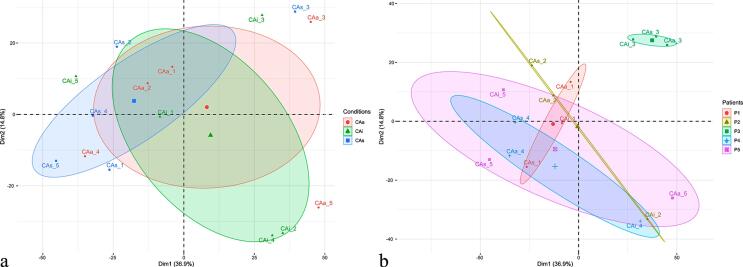
Variability in microbial composition among conditions (Fig.1a), and patients (Fig.1b) (R factoextra). The PCA plot Fig. 1.a separates samples according to caries status. The PCA plot Fig. 1.b separates samples according to subjects. Fig. 1a: Red represents biofilm samples from active non-cavitated caries lesions (CAa); green corresponds to arrested non-cavitated caries lesions (CAi), and blue to sound tooth surfaces (CAs). Fig. 1b: Red corresponds to all samples of CA1 patient (P1); olive green corresponds to all samples of CA2 patient (P2); green corresponds to all samples of CA3 patient (P3); blue corresponds to all samples of CA4 patient (P4), and purple corresponds to all samples of CA5 patient (P5). Ellipses represent 95% confidence interval

Some variability in microbial composition was observed in relation to the patients and the clinical conditions of the tooth surface regarding diversity. Overall, according to the clinical status of the tooth surface, the PCA analysis reveals a central core and an overlap of all conditions (active, arrested, and sound surfaces) (Figure 1a). At the individual level, patient P3 presented a distinct active microbial composition compared to the other patients, elucidating the importance of intra-individual conditions in biofilm composition (Figure 1b). Patients P1, P2, P4, and P5 have similar microbial profiles. Both P3 and P1 presented less variable microbial compositions since the distance of the different surfaces is closer. The microbial composition has a wide distribution, being more pronounced in patients P4 and P5.

**Figure 2:**
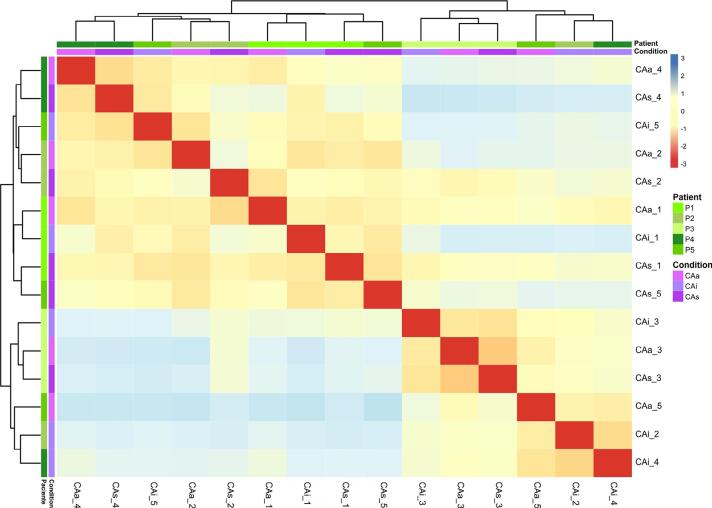
Euclidean distance plot. Green shades represent patients (patient 1 (P1); patient 2 (P2); patient 3 (P3); patient 4 (P4); and patient 5 (P5)). Purple shades represent tooth surface clinical condition (active non-cavitated surfaces – CAa; arrested non-cavitated surfaces – CAi; and sound surfaces - CAs)

Two main clusters can be identified in the heatmap generated by Euclidean distance analysis; all patients and conditions were mixed (Figure 2). Different surfaces from patient 1 presented a similar composition since all conditions were assembled in a cluster. Patient 3’s surfaces share not only the same cluster, but also the same branch, reinforcing their similar microbial profiles (as shown in Figure 1b). The metatranscriptome data for all genes are similar and present a high correlation coefficient (above 0.6) in all conditions (data not shown). These results show functional redundancy among the distinct clinical statuses of the tooth surface.

**Figure 3:**
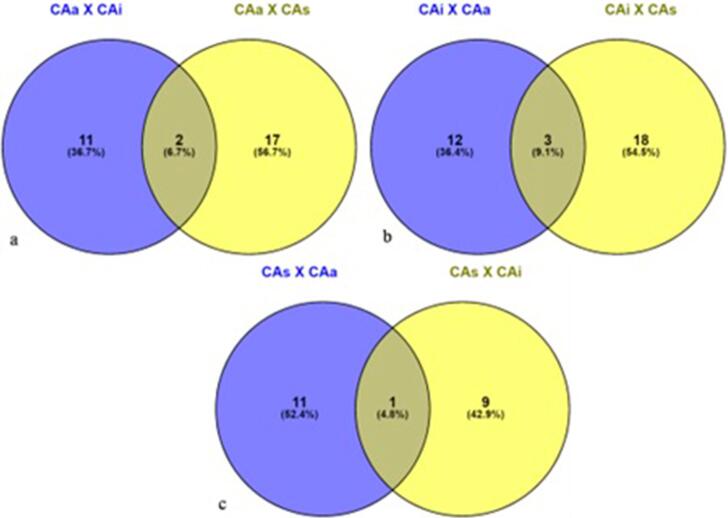
Venn diagram depicting the overlap between the differential expressed genes of the three different biofilm collected tooth surfaces (CAa – non-cavitated caries lesions surfaces; CAi – arrested non-cavitated lesions surfaces; CAs – sound tooth surfaces). Fig 3a represents genes up-regulated in CAa related to CAi (blue – 13 genes) and related to CAs (yellow – 19 genes); Fig 3b represents genes up-regulated in CAi related to CAa (blue – 15 genes) and related to CAs (yellow – 21 genes); Fig 3c represent genes up-regulated in CAs related to CAa (blue – 11 genes), related to CAi (yellow – 9 genes). Intersections between blue and yellow diagrams mean common differentially expressed genes between the comparisons

Thirty-two genes are up-regulated in CAa when compared to CAi or CAs. Two genes are commonly up-regulated in CAa compared to CAi and CAs: *oprN* codes for an outer membrane protein, and K06883 is related to an uncharacterized protein (Figure 3a). These genes are strongly related to active-caries tooth sites. The resistance gene (*oprN*) codes for multidrug resistance or protection against toxic components such as nitric oxide. Thirty-three genes are up-regulated in CAi. Three genes are commonly up-regulated in CAi compared to CAa and CAs: *rtxA* (Signaling), *MTHFS* (Biosynthesis of metabolites) and *FTO* (Genetic information) (Figure 3b). Twenty-one genes are up-regulated in CAs. One gene is commonly up-regulated in CAs compared to CAa and CAi, K07006 (uncharacterized function) (Figure 3c). The differential expression of the up-regulated genes from Figure 3 in each condition is detailed in the Supplementary Tables (1 to 3).

**Figure 4:**
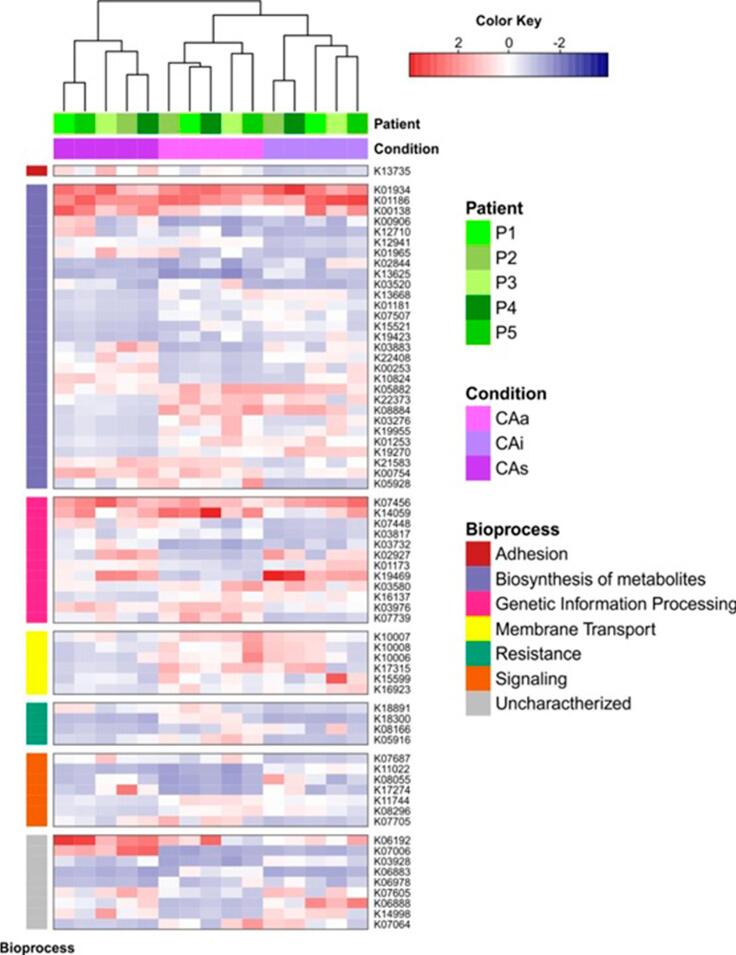
Heatmap representing all genes up-regulated in one or more conditions and patient origin. Genes are grouped by bioprocess. P1 (patient 1); P2 (patient 2); P3 (patient 3); P4 (patient 4);P5 (patient 5). Conditions: CAa (non-cavitated caries lesions surfaces of caries-active patients); CAi (arrested non-cavitated lesions surfaces of caries-active patients); CAs (sound tooth surfaces of caries-active patients). Blue indicates negative correlation and red indicates positive correlation

Up-regulated genes in CAa are related to 5 major functions (Figure 4; Supplementary Table 1): resistance (*oprN*; *YHB1*; *lmrC*), biosynthesis of metabolites (E2.1.1.95; *coxL*; *abgB*; K08884; *waaR*; *pimB*; *EPHX1*; *larA*), genetic information processing (*ELP3*; *rimL*; *int*; *ybaK*; *nemR*; hepA), signaling (*lytT*; *rcsB*; *tqsA*; *sixA*) and membrane transport (*gtsA*; *gluA*; *gluC*; *qrtT*; *gluD*). Three genes up-regulated in CAa have an unknown function (K06883; K07064; K06978). Some up-regulated genes in CAa were related to carbohydrates, or rare metabolites, indicating a more complex nutrient need for survival in an extreme environment. Signaling genes were up-regulated in CAa related to CAi (*lytT*; *rcsB*). Bacteria have several regulatory mechanisms in response to environmental changes, and the two-component system is one of them. These genes connect changes in input signals to changes in cellular physiological output. This function is essential for adaptation in an acidic environment.

Glycosyltransferases related to lipopolysaccharide synthesis are up-regulated in carious tooth surfaces (*waaR*; *rfaG*). Glycosyltransferase activity was up-regulated in CAi (*epsE*). The metabolism of myo-inositol was up-regulated in carious surfaces (*pimB*; *mshA*). Myo-inositol is a type of vitamin B and vital for the metabolism of yeasts and Archeas. Bacteria possess many kinases that catalyze the phosphorylation of proteins on diverse amino acids, including arginine, cysteine, histidine, aspartate, serine, threonine, and tyrosine. These protein kinases regulate different physiological processes in response to environmental modifications.^22^

Up-regulated genes in CAi are related to 5 major functions (Figure 4; Supplementary Table 2): Signaling (*rtxA*; *B2M*; *tqsA*), biosynthesis of metabolites (*MTHFS*; *FTL*; *dac*; *NEU1*; K08884; *ND5*; *hxpA*; *rfaG*; *EPHX1*; *pimB*; *mshA*; *epsE*; *mgtC*; *xynA*; *adh2*; *E1.1.1.91*), genetic information processing (*FTO*; *UBA52*; *ENDOG*; *mutS2*; *nemR*), membrane transport (*gtsA*; *qrtT*; *thiX*), and resistance (*mmr*). Five genes up-regulated in CAi have unknown function (K06888; *SURF1*; *yvaK*; *KRT2*; K07064). Metabolically, these genes up-regulated in CAi are related to biofilm maintenance.

Up-regulated genes in CAs are related to 5 major functions (Figure 5; Supplementary Table 3): Resistance (*lmrC*), genetic information processing (*FTO*; *SURF1*; *rhlB*; *UBA52*; *mrr*), signaling (*rcsB*; *S100A10*), biosynthesis of metabolites (*ND5*; *aceK*; *ivd*; *dac*; *nikE*; *aldB*; *novU*; *PCCA*; *grdG*; *bshA*), and adhesion (*yeeJ*). Two genes up-regulated in CAs have an unknown function (K07006; pqiB). Interestingly, the gene L-malate (*bshA*) is up-regulated in CAs and is related to acid and salt resistance, sporulation, and resistance to electrophiles and thiol-reactive compounds. Overall, genes up-regulated on sound surfaces are more associated with basal biofilm metabolism than with microbial virulence traits.

## Discussion

According to the clinical status of the tooth surface, this study revealed metabolically active supragingival biofilm. Although in our study the sample was small, which was a limitation, it was possible to verify that the biofilms from sound, arrested, and active lesions are similar in composition and function. Figure 1 reveals an overlap of all conditions (active, arrested, and sound surfaces) in caries-active patient, suggesting that dysbiosis is occurring within the whole mouth. This should be considered for new caries prediction models. Therefore, in studies of naturally occurring oral biofilms, the pooling of biofilms from similar sites (preferably including carious surfaces) seems to be the best practice for investigating the microbiology of caries-active patients. Clinically, this implies that healthy and arrested surfaces in caries-active individuals may be at microbiological risk of developing caries lesions. Longitudinal clinical studies have proven that the progression rate of non-cavitated arrested lesions was significantly higher in caries-active individuals than in caries-inactive individuals.^11,23^

The PCA analysis (Figure 1b) shows that patient P3 has a distinct microbial composition compared to the other subjects analyzed. According to Zaura, Pappalardo, Buijs^24^ (2021), clinical conditions are important during the results translation of a metagenomic study.^24^ Patient 3 (P3) has different oral conditions from the other active subjects in this study. This supragingival biofilm sample was collected from sixty-year-old women who wore removable dental prostheses (Figure 3). It is possible to see that, for P3, all conditions are present in the same branch of the dendrogram, showing similar microbial profiles (as shown in Figure 1b). Therefore, participant selection should be made carefully, considering their clinical and oral conditions, to optimize the quality of clinical studies in the oral microbiome.^24^

All the dental surfaces analyzed in this study showed a functional redundancy in biofilms (Figure 2). In addition to an expected difference in microbial composition,^10^ these samples showed a high positive correlation coefficient (above 0.6) in all conditions regarding functional profile. This functional redundancy is associated with the fact that multiple distinct microorganisms can perform similar metabolic functions.^25^ In our study, the biosynthesis of metabolites was the most expressed function associated with enamel dental caries. These findings follow the literature, which shows that the functional profile of caries-associated bacterial communities is related to acid stress tolerance and dietary sugar fermentation.^8^ Other studies present a similar mycobiome^26^ composition and metagenomic profile^27^ from distinct surfaces in the same patient.

Regarding differential gene expression (Figure 3), some genes (*rcsB* - CAa vs CAi and CAs vs CAi, K08884 - CAa vs CAi and CAi vs CAs) are related to biofilm formation and are present in all surfaces of caries-active individuals. Biofilm formation is essential for caries disease development on smooth surfaces. The microorganisms need to be able to adhere to the tooth hard surface before lesion formation and during caries progression.

Special functions related to the surfaces of caries-active patients need to be highlighted, such as those regarding ABC transporters. The ABC transporters constitute a superfamily of membrane proteins responsible for the ATP-powered translocation of many substrates across membranes. These sugar transporters are essential for carbohydrate uptake and are therefore over-expressed in caries-active and arrested samples (*gtsA* - CAa vs CAs, CAi vs CAs, *qrtT* - CAa vs CAs, CAi vs CAs), suggesting higher sugar intake rates.^28^

The *tqsA* gene (CAa vs CAs, CAi vs CAs) has its function related to the AI-2 transport protein. AI-2 plays a vital role in virulence-related gene expression.^29^ The *LuxS*/autoinducer-2 (AI-2) quorum sensing system regulates mixed-species biofilms, and AI-2 is proposed as a universal signal for the interaction between bacterial species.^30^ AI-2 is also involved in glucosyltransferase gene regulation required for sucrose-dependent biofilm formation. Our results agree with previous studies,^31^ highlighting that the AI-2 gene plays an essential role in the formation of caries-related oral biofilm.

Similar to the results obtained from RNA-seq of *Candida albicans* in root caries samples,^32^ the inositol genes (*pimB* - CAa vs CAs, CAi vs CAs, *mshA* - CAi vs CAs) were also up-regulated in caries-active conditions. Genes *pimB* and *mshA* are related to the ability to survive in an extreme environment^33^ with several stress factors (low pH, carbohydrate viability, for example), such as those genes found in cavitated root caries lesions.

*EPHX1* is a gene up-regulated in caries-active and arrested surfaces (CAa vs CAs, CAi vs CAs) related to xenobiotics degradation and metabolism**.** Microorganisms have a remarkable catabolic potential, with genes, enzymes, and degradation pathways involved in the biosynthesis of different metabolites**.** Microorganisms potentially use xenobiotic contaminants as carbon or nitrogen sources to sustain their growth and metabolic activities. Diverse microbial populations survive in harsh contaminated environments,^34^ exhibiting significant biodegradation potential to degrade and transform chemicals in ultra-processed food, antibiotics, and oral hygiene products. The relationship of xenobiotics degradation and dental caries should be further explored in future studies.

The *YHB1* gene (CAa vs CAi) is a nitric oxide dioxygenase. Nitric oxide has antimicrobial properties^35^ that suppress the growth of other species,^36^ providing resistance to plaque accumulation.^37^ Salivary nitrate concentrations could generate nitric oxide concentrations that decrease biofilm formation by susceptible species,^38^ providing resistance and recovery from plaque accumulation. This is in line with our results, since nitric oxide dioxygenase is up-regulated in CAa enamel. Copper-containing nitrite reductase was also considered a biomarker related to carious biofilm in another study.^28^

Lactate racemase (*larA*) was up-regulated in CAa *vs* CAs. Racemases catalyze the inversion of stereochemistry in biological molecules, giving the organism the ability to use both isomers. Lactic acid (L- and D-isomers) is an essential and versatile compound produced by microbial fermentation.^39^ The use of lactate in cariogenic biofilm is a crucial virulence factor.

Regarding genes up-regulated in arrested surfaces of caries active individuals (CAi), the *FTL* gene (CAi vs CAa) encodes the light subunit of the ferritin protein. Ferritin is the major intracellular iron storage protein in prokaryotes. It is composed of 24 subunits of heavy and light ferritin chains. Variation in ferritin subunit composition may affect the rates of iron uptake and release in different tissues. A primary function of ferritin is the storage of iron in a soluble, nontoxic state. Iron is typically a limiting factor in bacterial growth.^40^ Reduced iron availability in the oral cavity creates a constant competition between bacteria and human cells for this essential nutrient.^28^

The *KRT2* gene (CAi vs CAa) is related to keratin degradation. Interestingly, another study that carried out metaproteomics of oral biofilm also found proteins related to epithelial cells, such as keratin, in the samples. Although the samples were collected only from tooth surfaces, high amounts of epithelial-related proteins (keratin, cell junctions, desmosomes) were found. Since oral epithelia are continuously shed from the mucosa, human cells could be found in biofilm. The hypothesis presented by these authors, to be confirmed in other studies, is that epithelial cells may serve as vehicles for microbial transport into the biofilm and also serve as a nutrient source for the growing biofilm.^28^ Since we found a keratin-related gene up-regulated in the biofilm, we agree with the feasibility of those findings.

The gene *dac* (CAi vs CAa, CAs vs CAa) has its function related to diacetylchitobiose deacetylase. This gene deacetylates the non-reducing end of diacetylchitobiose (GlcNAc2) and is probably involved in chitin degradation, part of Glycan degradation. Interestingly, diacetylchitobiose is a moiety of the sugar chain of human salivary amylase.^41^ This gene could be associated with human amylase as the energy source for microorganisms. In a proteomic study of biofilm related to caries, N-acetylglucosamine-6-phosphate deacetylase, an enzyme that degrades this amino-sugar present in a wide variety of macromolecules coming from saliva and gingival crevicular fluid, was considered a biomarker of health.^28^ The presence of this gene in a sound site biofilm concurs with the current literature.

## Conclusions

Metatranscriptome data from subjects with caries activity have shown that biofilms from sound, arrested, and active lesions are similar in composition and function. This suggests that a biofilm pool, rather than individual lesions, should be used for metabolic and functional studies of dental caries. Clinically, this implies that healthy and arrested surfaces in caries-active individuals may be at microbiological risk of developing caries lesions.
